# Development, Characterization, and Evaluation of α-Mangostin-Loaded Polymeric Nanoparticle Gel for Topical Therapy in Skin Cancer

**DOI:** 10.3390/gels7040230

**Published:** 2021-11-24

**Authors:** Shadab Md, Nabil A. Alhakamy, Thikryat Neamatallah, Samah Alshehri, Md Ali Mujtaba, Yassine Riadi, Ammu K. Radhakrishnan, Habibullah Khalilullah, Manish Gupta, Md Habban Akhter

**Affiliations:** 1 Department of Pharmaceutics, Faculty of Pharmacy, King Abdulaziz University, Jeddah 21589, Saudi Arabia; nalhakamy@kau.edu.sa; 2 Center of Excellence for Drug Research & Pharmaceutical Industries, King Abdulaziz University, Jeddah 21589, Saudi Arabia; 3 Mohamed Saeed Tamer Chair for Pharmaceutical Industries, King Abdulaziz University, Jeddah 21589, Saudi Arabia; 4 Department of Pharmacology & Toxicology, Faculty of Pharmacy, King Abdulaziz University, Jeddah 21589, Saudi Arabia; taneamatallah@kau.edu.sa; 5 Department of Pharmacy Practice, Faculty of Pharmacy, King Abdulaziz University, Jeddah 21589, Saudi Arabia; Salshehri1@kau.edu.sa; 6 Department of Pharmaceutics, Faculty of Pharmacy, Northern Border University, Rafha 91911, Saudi Arabia; mmujtaba@nbu.edu.sa; 7 Department of Pharmaceutical Chemistry, College of Pharmacy, Prince Sattam Bin Abdulaziz University, Al-Kharj 11942, Saudi Arabia; y.riadi@psau.edu.sa; 8 Jeffrey Cheah School of Medicine and Health Sciences, Monash University, Subang Jaya 47500, Malaysia; ammu.radhakrishnan@monash.edu; 9 Department of Pharmaceutical Chemistry and Pharmacognosy, Unaizah College of Pharmacy, Qassim University, Unaizah 51911, Saudi Arabia; h.abdulaziz@qu.edu.sa; 10 Department of Pharmaceutical Sciences, School of Health Sciences, University of Petroleum and Energy Studies (UPES), Dehradun 248007, India; manish.gupta@ddn.upes.ac.in; 11 School of Pharmaceutical and population Health Informatics (SoPPHI), DIT University, Dehradun 248009, India

**Keywords:** α-Mangostin, polymeric nanoparticle, skin cancer, gel, Behnken design, MTT assay, antioxidant assay

## Abstract

The aim of this study was to prepare and evaluate α-mangostin-loaded polymeric nanoparticle gel (α-MNG-PLGA) formulation to enhance α-mangostin delivery in an epidermal carcinoma. The poly (D, L-lactic-co-glycolic acid) (PLGA) nanoparticles (NPs) were developed using the emulsion–diffusion–evaporation technique with a 3-level 3-factor Box–Behnken design. The NPs were characterized and evaluated for particle size distribution, zeta potential (mV), drug release, and skin permeation. The formulated PLGA NPs were converted into a preformed carbopol gel base and were further evaluated for texture analysis, the cytotoxic effect of PLGA NPs against B16-F10 melanoma cells, and in vitro radical scavenging activity. The nanoscale particles were spherical, consistent, and average in size (168.06 ± 17.02 nm), with an entrapment efficiency (EE) of 84.26 ± 8.23% and a zeta potential of −25.3 ± 7.1 mV. Their drug release percentages in phosphate-buffered solution (PBS) at pH 7.4 and pH 6.5 were 87.07 ± 6.95% and 89.50 ± 9.50%, respectively. The release of α-MNG from NPs in vitro demonstrated that the biphasic release system, namely, immediate release in the initial phase, was accompanied by sustained drug release. The texture study of the developed α-MNG-PLGA NPs gel revealed its characteristics, including viscosity, hardness, consistency, and cohesiveness. The drug flux from α-MNG-PLGA NPs gel and α-MNG gel was 79.32 ± 7.91 and 16.88 ± 7.18 µg/cm^2^/h in 24 h, respectively. The confocal study showed that α-MNG-PLGA NPs penetrated up to 230.02 µm deep into the skin layer compared to 15.21 µm by dye solution. MTT assay and radical scavenging potential indicated that α-MNG-PLGA NPs gel had a significant cytotoxic effect and antioxidant effect compared to α-MNG gel (*p* < 0.05). Thus, using the developed α-MNG-PLGA in treating skin cancer could be a promising approach.

## 1. Introduction

Skin cancer is the most common malignancy affecting a large percentage of people in many countries worldwide [1]. The skin has a large surface area, subjected to several harmful environmental factors such as chemicals, toxic substances, and ultraviolet rays, which apparently can cause abnormal growth of cells and eventually cancers [2]. The skin cancer type is identified based on the cellular origin, either melanoma skin cancer (MSC) or nonmelanoma skin cancer (NMSC) [3]. Melanoma is the least common type, but it is responsible for a high number of deaths among patients with skin cancer [4]. However, the incidence of NMSC is higher, and it includes two types: basal cell carcinoma (BCC) and squamous cell carcinoma (SCC) [5]. The cancerous cells may travel to a distant part of the body and metastasize to develop melanoma, which may remain undiagnosed and subsequently not be treated timely [6]. 

Chemotherapy is considered the standard approach for destroying skin cancerous cells. However, it has several undesirable outcomes in treating skin cancer, including reduced systemic effects and bioavailability [7]. In addition, chemotherapeutic agents cannot differentiate between normal and cancerous cells. Thus, nanotechnology emerges as a remarkable solution for drug delivery [8]. This technology utilizes polymer nanoparticles (NPs) that allow the primary drug to reach the targeted region using a delivery vehicle that is versatile, biocompatible, biodegradable, and nanosized. In cancer, prolonged circulation of NPs enriches drug availability to the tumor tissues through direct permeation and diffusion to the cells or enhanced permeation and retention effects [9,10,11,12]. The transport of NPs across distinguished skin layers may face difficulty in permeation and penetration, probably due to the hard and horny multilayer of stratum corneum, followed by dermis barriers. The NPs may transport the drug through the skin’s appendages, such as hair follicles and sebaceous and sweat glands. Hair follicles may act as reservoirs for polymeric NPs due to their deeper penetration and release of drugs into different skin layers, including the stratum corneum. Particles smaller than 10 nm are easily excreted out and phagocytized. NPs are internalized in the target cells via a series of biological events, including early and late endosome, thereby fusing with the lysosome, and diffusing into the cytoplasm, and finally destined in the nucleus of the cells. They may also internalize into cells through pinocytosis or phagocytosis process [13]. The transport of NPs into the tumor region mainly relies on physicochemical features, including size, surface, topography, and nano-biointeraction in the biological milieu [14].

A gel is a three-dimensional swellable polymeric network with hydrophilic groups cross-linked by a strong force of interaction that can incorporate both hydrophilic and lipophilic drugs. It has excellent biocompatibility, biodegradability, flexibility, drug stability, and controlled drug release. Moreover, it has a tendency to retain a large quantity of aqueous fluid without dissolving onto them and is widely explored in drug delivery and biomedical engineering. The polymeric network in the gel system plays a significant role in determining physicochemical properties and hydrophilicity, and controlling particle size; small pores in the polymeric network enables the accommodation of small molecules [15].

In the current era, bioactive agents from natural sources have been utilized enormously in pharmaceutical and nutraceutical preparations due to their wide range of biological activity. Interestingly, incorporating nanotechnology and phytomedicine may provide alternative treatment strategies for cancer as these bioactive agents normally cause no harm and most of them only exhibit minimal side-effects and toxicity at appropriate doses [16]. The activity of the phytoconstituents can be enhanced by encapsulating them in polymeric nanocarriers. For this purpose, several phyto-based NPs are designed and developed for site-specific targeting to improve drug therapeutic efficacy and potency.

Polymeric NP/gel is composed of poly D,L-lactic-co-glycolic acid (PLGA) a biodegradable linear copolymer produced by polymerization of monomeric units of lactic and glycolic acid that leads to nontoxic degraded products, i.e., water and carbon dioxide. The degradation rate of PLGA depends on the lactic acid/glycolic acid ratio and the molecular weight of the polymer. Polyglycolic acid has low water solubility due to its crystalline hydrophilic nature. On the other hand, the polylactic acid counterpart is somehow a stiff, hydrophobic polymer with low mechanical strength [17,18].

**Figure 1:**
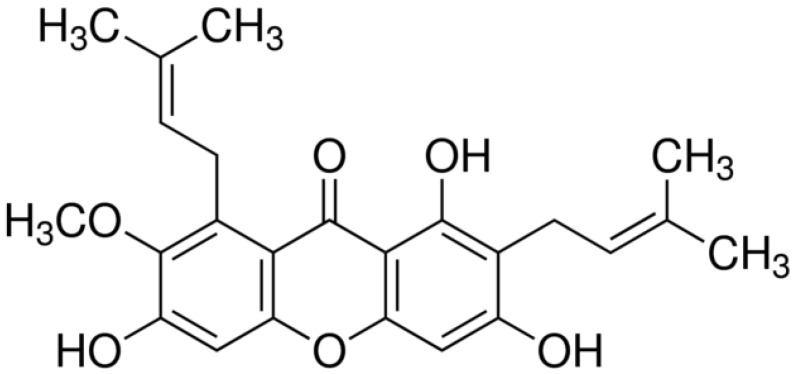
Chemical structure of α-MNG.

Alpha-Mangostin (α-Mangostin) is a natural xanthin found in the bark and fruit of the tropical plant *Garciniamangostana* Linn. The fruit is dark and reddish, with a soft, juicy, sweet, acidic pulp. The pericarp of the fruit is used in traditional medicine to treat various disorders, including skin infections. The main constituent of α-mangostin is xanthone glycoside, 1,3,6,7-tetrahydroxyxanthone-C2-β-d glucoside (Figure 1) [19,20]. It has several biological activities such as antioxidant, anticancer, anti-inflammatory, antiaging, antiviral, and antidiabetic effects [21,22]. The antioxidant properties are attributed to polyphenols, which combat free radicals (ROS) generation, thereby preventing DNA and cell/tissue damage. The generated free radicals require detoxification and should be eliminated from the body to avoid the occurrence of cancers. 

The current study aimed to design, optimize, and develop α-MNG-PLGA NPs and analyze the skin permeation and retention of the drug from nanocarriers within the distinguished layer of animal skin [23,24]. The formulation was tested in a melanoma cell line for its anticancer and antioxidant properties. Moreover, a stability study of the developed formulation was evaluated. 

## 2. Results and Discussion

### 2.1. Optimization 

A Box–Behnken experimental design was utilized for optimizing the formulation. It is a commonly used design as it costs less to run the experiment within the stipulated period, and it is a more efficient optimization technique than the conventional optimization procedure. The independent variables were polymer concentration, surfactant concentration, and sonication time (ST) and their impact effects were studied on critical quality attributes, such as particle size (PS), PDI, and entrapment efficiency (EE) of PLGA NPs. The dependent variables were used in three levels: low (−1), medium (0), and high (+1); accordingly, at these levels, the polymer concentrations (X1) were 2.5 mg (low); 3.00 mg (medium), and 3.50 mg (high); surfactant concentrations (X2) were 1% *w*/*v* (low), 1.75% *w*/*v* (medium), and 2.50% *w*/*v* (high); ST (X3) were 4 min (low), 8.00 min (medium), and 12.00 min (high), respectively. The low (−1) and high (+1) ranges of the independent variables were selected based on a preliminary examination and were found to be suitable for robust and consistent formulation. At the concentration of X_1_ > 3.00% *w*/*v*, the PS was too large; below 2.5% *w*/*v*, the EE and drug loading were too low. Furthermore, ST > 12.00 min caused a further reduction in PS, which led to surface charge generation and ultimately caused the reaggregation of particles, which further impeded the stability of the formulation. ST < 4.00 min did not appropriately reduce PS to the desired size. The independent variables levels (low, medium, and high) and corresponding responses (minimize or maximize) are given in Table 1. The experimental design generated 17 distinguished formulations of varying compositions and responses (Table 2). The five replicas as shown in Table 2 were software-generated and an in vitro investigation revealed that they had varied particle size (nm) (Y_1_); PDI (Y_2_); and entrapment efficiency (%) (Y_3_) but variation was insignificant. Similar results were observed in a previous study [25,26,27,28,29,30].

**Figure 2:**
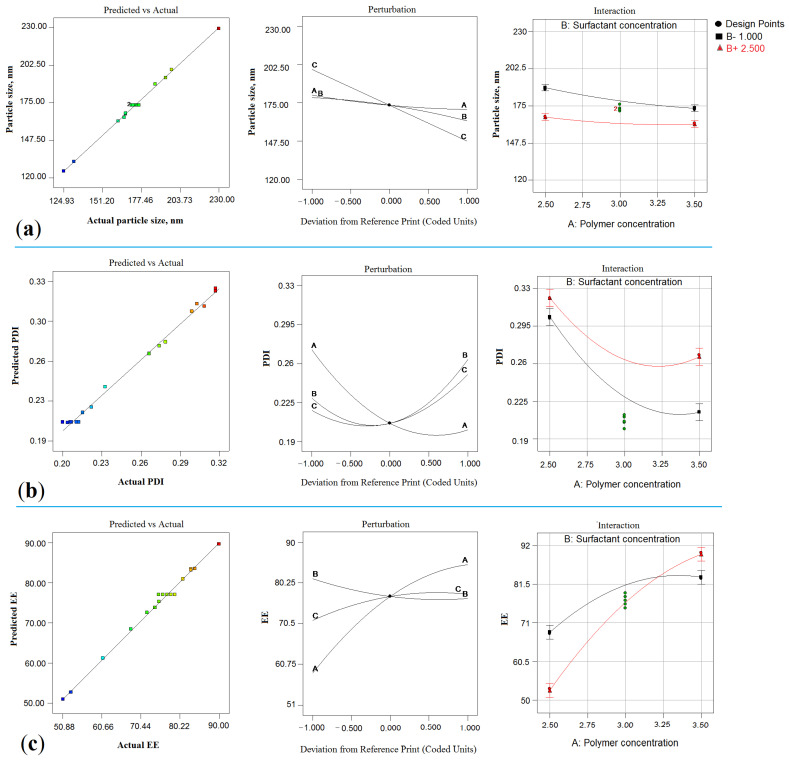
Model diagnostic plot representing linear correlation plot between predicted vs. actual, perturbation chart and interaction plot for responses particle size, Y_1_ (**a**); entrapment efficiency, Y_2_ (**b**) and PDI, Y_3_ (**c**). (A = X_1_ = Polymer concentration; B = X_2_ = Surfactant concentration; and C = X_3_ = Sonication time).

**Figure 3:**
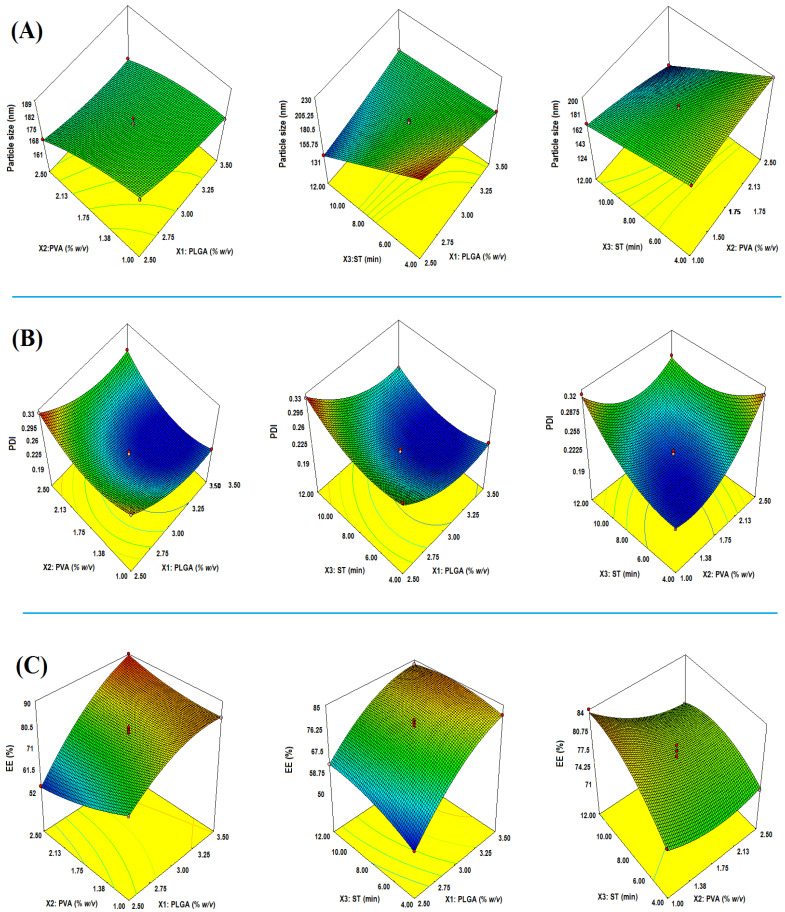
3-D response surface morphology (**A**–**C**) exemplifying comparative impact of input attributes; PLGA (% *w/v*), PVA (% *w/v*), and ST (min) on critical quality attributes; particle size (**A**), PDI (**B**) and entrapment efficiency (**C**).

The linear correlation plots between predicted vs. actual, perturbation chart, and interaction plot have shown a good fit for the PS, PDI, and EE responses, as shown in Figure 2a–c. The 3D response surface plot suggesting the comparative impact of factors on PS, PDI, and EE is shown in Figure 3A–C. The best-fitted model with a coefficient of correlation (R^2^)~1 was selected for further study after the data fitted to a different model. Models with a good fit for every response were taken with ANOVA by estimating F values. Table 3 shows the results of the regression analysis for the selected responses, PS (Y_1_), PDI (Y_2_), and EE (Y_2_), for adjustment into the polynomial equation. The optimum values of different responses, PS, PDI, and EE, were obtained using the point prediction method on desirability criteria.

### 2.2. Effect on Particle Size (Y_1_)

The effects of PLGA, PVA, and STon size are explicated by the quadratic equation as follows:

PS = +173.16 – 5.12 × X1 – 8.37 × X2 – 25.75 × X3 + 2.50 × X1 × X2 + 22.75 × X1 × X3 – 11.25 × X2 × X3 + 2.14 × X1^2^ – 2.86 × X2^2^ (1)

Model F-value of 334.52 entailed that the model was significant. Herein, X1, X2, X2, X1X2, X1X3, X2X3, X1^2^, and X2^2^ are significant model terms. The lack of fit (F-value) of 0.76 implies that the model was insignificant. “Pred R^2^” of 0.9970 is in reasonable agreement with “Adj R^2^” of 0.9940, as shown in Table 3. The adequate precision (signal-to-noise ratio) of 79.146 indicates an adequate signal. Therefore, the model was qualified for navigating the design space. In the quadratic equation, positive sign (+) indicates the synergistic effect and negative sign expressed antagonistic effect, explaining the individual, combined, and quadratic effects [28,29,31]. The effects of all the input attributes (X1, X2, and X3) on PS are shown in the 3D plot (Figure 3A).

#### 2.2.1. Impact of PLGA

The PS significantly influences the fate of NPs in a biological fluid, such as the processes of dissolution, absorption, biodistribution, and cellular uptake. The PLGA concentration had a lesser negative impact on the PS of NPs. The ranges of PS were 125.00 ± 10.01 to 230.00 ± 33.76 nm. The PS at low PLGA concentration (2.5% *w/v*) was observed at 132 ± 12.67 nm (NP17) and 167 ± 17.12 nm (NP10). By increasing the PLGA concentration to 3.00% *w/v*, the maximum increase in PS was observed to be 176 ± 19.04 nm, as seen in formulation NP1 and 194 ± 24.98 nm in the case of formulation NP13. Furthermore, an increase in PLGA concentration reduced PS to 162 ± 14.03 nm (NP12), probably due to the interaction effect with a higher concentration of PVA (2.5% *w/v*) in this formulation composition. The results are consistent with those of the previously reported method [32].

#### 2.2.2. Impact of PVA

The surfactant plays a significant role in controlling NPs size and generally has a negative impact. The increasing PVA (1.75% *w/v*) concentration reduced the PS to 132 ± 12.67 nm in the NP17 formulation. Furthermore, an increase in PVA concentration (2.5% *w/v*) led to a blending effect on the PS, namely, reduced PS to the maximum value of 125 ± 10.01 nm as seen in the NP6 formulation, whereas another formulation NP9 has shown increased PS up to 198 ± 19.62 nm. At a higher surfactant concentration (2.5% *w/v*), the PS increased to 198 ± 19.62 nm due to a significant reduction in the surface area, which led to the reagglomeration of nanoparticles. The surfactant was incorporated into the formulation to reduce the PS, prevent agglomeration, coalesceparticles, and stabilize the nanodroplets during the processing of the emulsion system [30,33]. The surfactant molecules stabilize the nanosystem by aligning themselves at the nanodroplet surface and reduce interfacial tension by lowering free energy [34]. 

#### 2.2.3. Impact of Sonication Time (ST)

The ST had a strong negative impact on PS. At low ST (4 min), bigger PSs were 198 ± 19.62 nm and 230 ± 33.76 nm in formulations NP9 and NP7, respectively. Further increasing ST (8 min) led to a maximum PS reduction to 162 ± 14.03 nm, as seen in the formulation NP12. Increasing the ST to 12 min yielded reduced PS of 132 ± 12.67 nm and 125 ± 10.01 nm in formulations NP17 and NP6, respectively. The subjecting probe sonication to NPs dispersion for optimal time increases the collision frequency, which disintegrates the large particles and agglomerates into monodisperse and homogeneous dispersion [35].

### 2.3. Effect on PDI (Y_2_)

The derived quadratic equation for PDI is given as follows.

PDI = +0.21 – 0.036 × X1 + 0.017 × X2 + 0.016 × X3 + 8.500E − 003 × X1 × X2 – 7.250 – 003 × X1 × X3 – 0.035 × X2 × X3 + 0.03 × X1^2^ + 0.040 × X2^2^ + 0.028 × X3^2^ (2)

The model F-value of 132.56 entails that the model was significant. Herein, X1, X2, X3, X1X3, X2X3, and X3^2^ are significant model terms. The lack of fit F-value of 1.41 entails that it was insignificant (*p* = 0.3622). “Pred R^2^” of 0.9942 was in reasonable agreement with “Adj R^2^” of 0.9967, as shown in Table 3. The model diagnostic plot representing a linear correlation between predicted vs. actual, perturbation chart, and interaction plot for response PDI is shown in Figure 2b. The impact of all the input attributes (X1, X2, and X3) on PDI is shown in the 3D plot (Figure 3B). 

#### 2.3.1. Impact of PLGA

As illustrated in the response surface curve, the increasing polymer has a negative influence on PDI. At low PLGA concentration (2.50% *w/v*) PDI was recorded as 0.302 ± 0.03 (NP2), 0.321 ± 0.02 (NP10), and 0.321 ± 0.05 (NP17), respectively. Further, by increasing PLGA concentration to (3.00% *w/v*), PDI decreased as to 0.276 ± 0.05 (NP6); 0.212 ± 0.03 (NP16); and 0.206 ± 0.01 (NP5), respectively. However, at a higher concentration of PLGA (3.5% *w/v*), PDI in some formulations was raised to 0.268 ± 0.02 (NP12), may be due to the large polymer concentration. The result is consistent with that of a previous report [36]. 

#### 2.3.2. Impact of PVA

At a low PVA concentration (1.00 *w/v*), PDI was 0.302 ± 0.03 (NP2) and 0.203 ± 0.07 (NP13), respectively. Furthermore, by increasing PVA concentration to 1.75% *w/v*, PDI was dropped to 0.199 ± 0.01 as seen in formulation NP11. However, at a higher concentration of the surfactant (2.5 % *w/v*), the PDI slightly increased, probably due to the combined effect of PLGA. The results agree with those of previous work [37]. As per the polynomial equation, PVA has a less positive impact on PDI. The low PDI with narrow size distribution is considered effective and ideal for NPs in drug delivery. The low value of PDI indicates the homogeneity and uniform distribution of PS in the developed formulation. The international standard organization has stated that the PDI value <0.05 indicates monodisperse size distribution, whereas value >0.7 is classified as a polydisperse size distribution [38].

#### 2.3.3. Impact of ST

ST has a less positive impact on the PDI of the formulation. The PDI of drug-loaded nanoparticles ranges between 0.199 ± 0.01 and 0.312 ± 0.06. The impact of independent variables on the NPs’ colloidal properties, especially the PS and PDI, is important as it determines the fate of NPs penetrating ability across cutaneous barriers [31]. The polydispersity measures the heterogeneity and size distribution of the sample as the process of agglomeration is high in nanoparticles. At ST of 4 min, the PDI was observed 0.306 ± 0.04 (NP9); at 8 min, further increasing of the blend effect was observed and PDI was reduced to 0.205 ± 0.02 in NP1 and 0.210 ± 0.04 in NP3 and increased to 0.302 ± 0.03 in NP2 and 0.321 ± 0.02 in NP10. At ST of 12 min, the PDI was 0.312 ± 0.06 in formulation NP15. The excess reduction in particles leads to reaggregation of particles, resulting in a larger particle diameter, which may be attributed to the increased PDI of the formulation. Despite the optimized formulation reported, the PDI value of 0.201 ± 0.01 (Table 4) indicates that the distribution of particle population has changed from a bimodal to unimodal shape [39,40]. 

### 2.4. Y3: Effect of on EE

The generated quadratic equation for EE is as follows:

EE = +76.98 + 13.00 × X1 − 2.37 × X2 + 3.13 × X3 + 5.50 × X1 × X2 – 2.00 × X1 × X3 − 1.75 × X2 × X3 − 5.37 × X1^2^ + 1.88 × X2^2^ − 2.61 × X3^2^ (3)

The model F-value of 119.42 entails that the model was significant. In this case, X1, X3, X1X2, X1X3, and X2X3 are significant model terms. The lack of fit of 0.16 implies that it was insignificant, while “Pred R^2^” of 0.9799 was in reasonable agreement with “Adj R^2^” of 0.9852 (Table 3). The adequate precision (signal-to-noise ratio), 39.150, indicates an adequate signal. Thus, the model is suitable for navigating the design space. The impact of all the input attributes (X1, X2, and X3) on EE is shown in the 3D plot (Figure 3C).

#### 2.4.1. Impact of PLGA 

The quadratic equation revealed that PLGA had a significant positive impact on EE of α-MNG in NPs. The EE of developed nanoparticles ranges from 51 ± 9.23% (NP7) to 90 ± 12.76% (NP12). Increasing the polymer increases the EE of a drug in NPs. The highest EE percentage reported for the formulation NP12 was 3.5% *w/v* of PLGA concentration. The EE mainly depends on the drug miscibility in the organic phase and drug-polymer interaction. Moreover, drugs solubilize in the polymeric solution due to enhanced emulsification properties [41]. The EE results agree with the findings regarding PLGA NPs reported in previous work [42].

#### 2.4.2. Impact of PVA

The above quadratic equation expressed the negative effect of PVA on EE. At a lower surfactant concentration (PVA, 1% *w/v*), the EEs were estimated to be 68 ± 6.12%, 74 ± 9.67%, and 84 ± 7.43%, respectively. Furthermore, when the PVA concentration is increased to 1.75% *w/v*, EE declines to 77 ± 9.56% in NP16 and 61 ± 7.12% in NP17, respectively. When the PVA concentration increased to 2.5% *w/v*, the least EE was recorded: 72 ± 11.92% in NP9 and 53 ± 4.47% in NP10. The main objective of adding PVA in the formulation is to reduce the PS and obtain the optimum size range. The PS reduction decreases the core volume of the polymeric shell, thus reducing the amount of drug entrapped. The concentration and type of surfactant are essential in the formulation of NPs, which plays a crucial role in stabilizing and protecting the nanodispersion system from coalescence and maintaining the homogeneity of the formulation [43]. 

#### 2.4.3. Impact of ST

ST has a less positive impact on the EE. The sonicating PLGA NPs for 4 min yielded EEs of 51 ± 9.23% in NP7 and 72 ± 11.92% in NP9. The maximum entrapment was 90 ± 12.76% by sonicating the formulation for 8 min (NP12), probably due to the combined effect of PVA and PLGA. 

### 2.5. Numerical Optimization

The numerical optimization technique was used to select the formulation with criteria of small PS, PDI, and high encapsulation efficiency. The selected optimized preparation indicates desirability closer to ~1, representing that the optimized method is vital. The selection of minimum PS provides more surface area for drug solubility and dissolution and, in the end, improves the bioavailability of the formulation [44,45]. The statistically optimized PLGA NPs are comprised of PLGA of 3.39% *w/v* and PVA of 1.82% *w/v* under probe ST of 8.79 min. The average observed PS was recorded at 168.06 ± 17.02 nm vs. the predicted PS of 150.87 nm. The average observed PDI value of PLGA NPs was 0.201 ± 0.01 vs. the predicted value of PDI 0.214. Similarly, the average EE of drugs was 84.26 ± 8.23% vs. predicted EE at 79.16%. The percentage errors in PS, PDI, and ST were 11.39, −6.07, and 6.44, respectively (Table 4). 

### 2.6. Characterization of α-MNG-PLGA NPs

**Figure 4:**
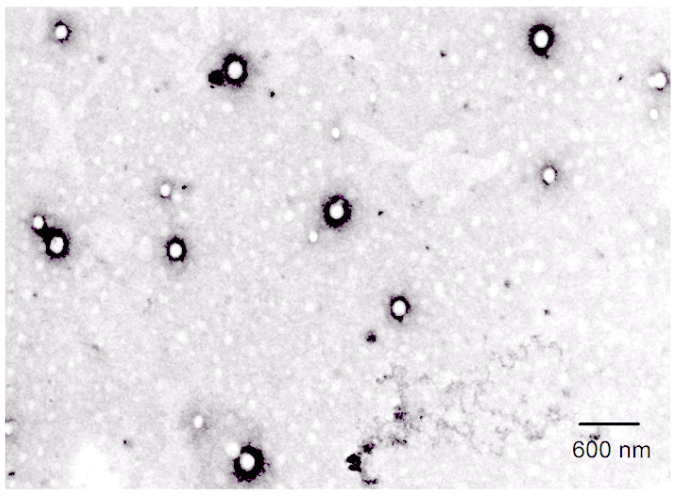
Transmission Electron microscopy image of Particle size α-MNG-PLGA NPs.

The transmission electron micrograph has shown that the optimized formulation of nanoparticles was consistent, spherical, well-dispersed, uniform in size, and deaggregated (Figure 4). The developed PLGA NPs possess high drug EE with reproducible size. The sonication of the polymeric system significantly controlled the PS using the preparation technique. The low value of PDI clearly indicates monodispersed nanoparticulate system and the negative surface charge of the nanoparticle was produced, which provides the colloidal stability and predicts the fate of NPs in vivo.

#### 2.6.1. Differential Scanning Calorimetry of α-MNG NPs

**Figure 5:**
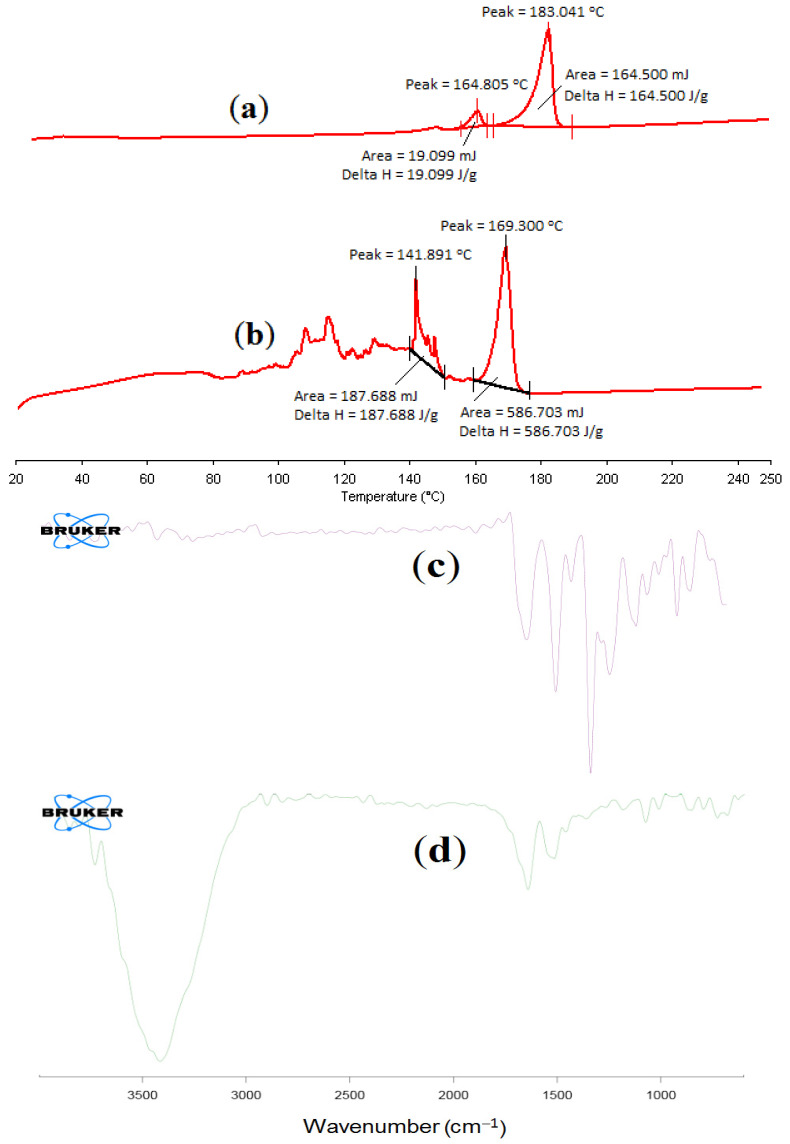
DSC thermogram showing melting point of plain α-MNG (**a**); and α-MNG-PLGA NPs (**b**). FT-IR of plain α-MNG (**c**) and α-MNG-PLGA NPs (**d**).

The DSC thermogram is a vital tool to identify the nature and alteration in the melting range of excipients in the physical mixture and PLGA NPs formulation. The thermal analysis results of plain α-MNG and α-MNG-PLGA NPs are shown in Figure 5A,B. The DSC thermogram revealed the strong endothermic peak of plain α-MNG at a melting point of 183.04 °C, indicating the crystalline structure of α-MNG alone. However, due to some interaction between polymer and α-MNG, the crystallinity of the drug is reduced, which is evident from the lowering of the melting point [46].

#### 2.6.2. FT-IR Spectral Analysis of α-MNG NPs

IR spectroscopic technique reveals the chemical stability of the entrapment in the NPs. The FT-IR spectra of α-MNG and α-MNG-PLGA NPs are shown in Figure 5c,d. These absorption bands of α-mangostin were also observed in the α-MNG-PLGA NPs. However, they appear to diminish to a flat level, suggesting that α-MNG is not interacting with other excipients in the formulation and is considerably entrapped within the NPs. Thus, the chemical stability of α-MNG in the NP is corroborated [46,47].

### 2.7. Drug Release and Kinetic Study

**Figure 6:**
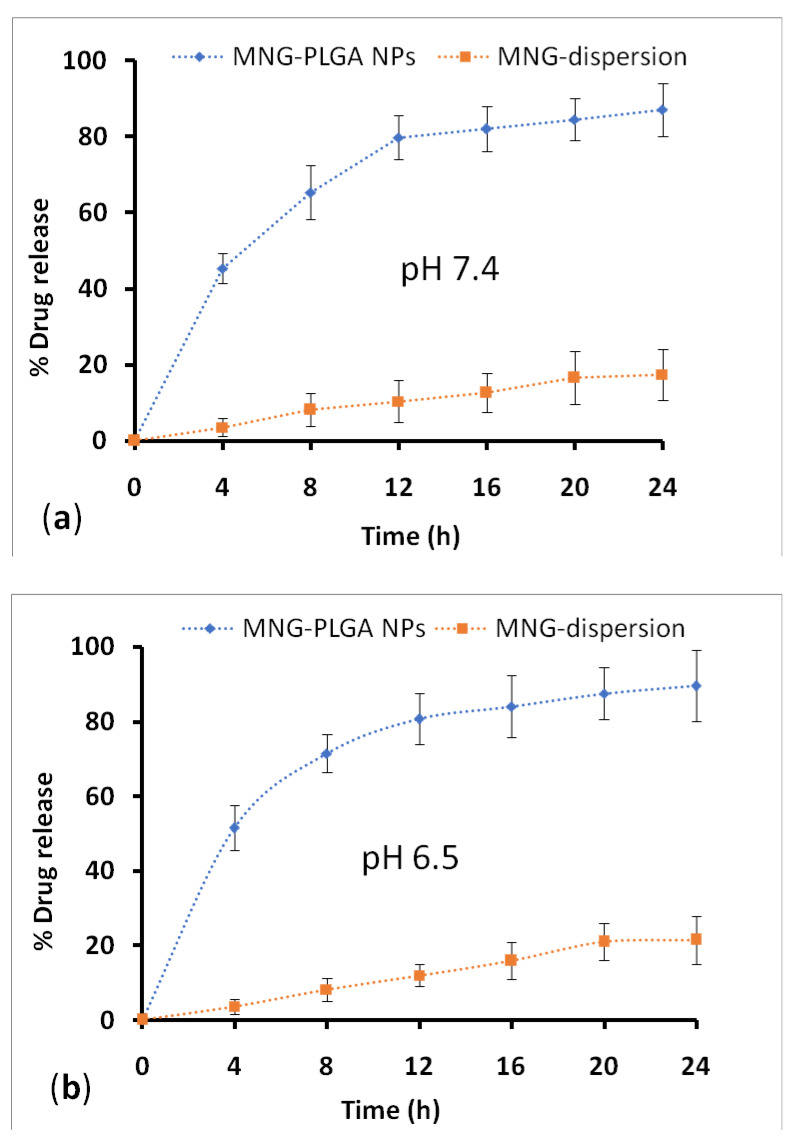
Percentage α-MNG release from α-MNG-PLGA NPs, and α-MNG-dispersion in PBS, pH 7.4 (**a**); and 6.5 (**b**), respectively.

The drug release profile of NPs at fixed time intervals is illustrated in Figure 6a,b. The results showed a biphasic release pattern; that is, drug release seems to burst in the early phase within 4 h (45.34%) accompanied by a sustained release profile over 24 h. The highest release of drug release from NPs at the end of the study (24 h) was 87.07 ± 6.95% in PBS at pH 7.4 compared to 17.43 ± 6.75% released from α-MNG dispersion. The minor drug release from drug dispersion may be due to the crystalline nature, poor solubility, and drug dissolution in PBS at a pH value of 7.4. Adversely, the drug release from polymeric NPs at pH 6.5 was 89.50 ± 9.50% vs. 21.48 ± 6.50% drug release from α-MNG dispersion. Moreover, drug release from polymeric NPs is significantly higher than plain α-MNG dispersion in both mediums (*p* < 0.05). However, the difference in the release under two different pH values was statistically nonsignificant (*p* > 0.05). The immediate drug release in contact with the dissolution medium from the polymeric surface or adsorbed drug and the controlled release from polymeric matrix comply with the biphasic drug release behavior from PLGA NPs. The drug release from PLGA NPs agreed with that reported in previous work [48]. The release profile of α-MNG from α-MNG-PLGA NPs was fitted to the different release kinetic models, for example, zero-order, Higuchi, first-order, and Hixson–Crowell models, to find the best suitable model. The regression value (R^2^) obtained from various models indicated that the best-fitted model for α-MNG-PLGA NPs was the Higuchi model (R^2^ = 0.9793). Furthermore, the underlying mechanism for drug release of α-MNG-PLGA NPs was assessed by applying the Korsmeyer–Peppas model, and the obtained *n* exponent valuewas 0.780 (between 0.5 and 0.89), which indicated that α-MNG release from NPs complied with non-Fickian diffusion [49]. The results displayed that α-MNG release from α-MNG-PLGA NPs is governed by a constant diffusion from the polymer matrix with the penetration of fluid, resulting in surface degradation and bulk erosion accompanied by coherent and ordered drug dissolution and succeeding release through diffusion in the acceptor compartment [50,51,52]. The drug release from α-MNG-PLGA NPs agrees with that reported in various studies on PLGA NPs and the research domain [53].

### 2.8. Gel Characteristics

The pH of the developed gel was slightly acidic, pH 6.72 ± 0.40, which did not cause skin irritation or erythema. The developed gel was of good appearance, consistency, and spreadability of 4.45 ± 0.45 cm within the acceptable range. The gel viscosity refers to the thickness of gel to flow over the skin. The viscosity of the α-MNG-PLGA NPs gel and the α-MNG gel was 3804.05 ± 102 and 3678.32 ± 302 cps, respectively. The gel is a stable colloidal particle with an excellent tunable and flexible surface, which can be easily manipulated with therapeutics. The soft and flexible architectureof the polymeric network makes penetration easy and improves the circulation time, thereby improving therapeutic drug delivery in vivo. The polymeric network can protect drug molecules and could release them intelligently in vivo. The gel characteristics in the current study were consistent with those of the previously reported carbopol gel [54].

### 2.9. Ex Vivo Skin Permeation 

**Figure 7:**
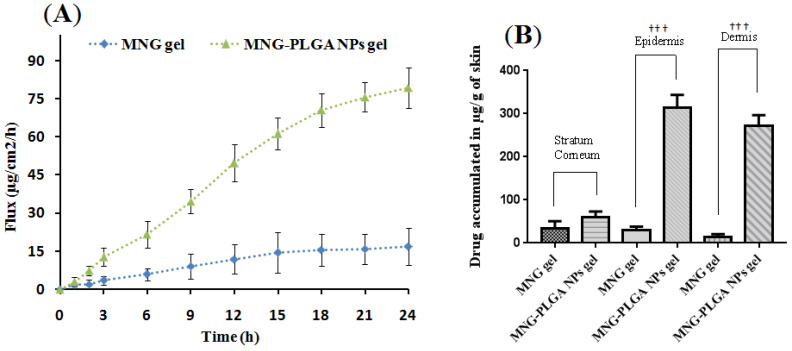
The steady state flux of α-MNG-PLGA NPs in skin permeation studies compared with flux of α-MNG gel (**A**). Concentration of drug retained in various layer of skin viz. stratum corneum, epidermis and dermis from α-MNG-PLGA NPs gel, α-MNG gel (Y), respectively (**B**). Data expressed as mean ± SD (*n = 3*) (††† *p* ≤ 0.01).

The drug diffusion and penetration study of α-MNG-PLGA NPs gel was carried out on rat skin using Logan’s assembly (Model FDC-6, Effem Technologies, New Delhi, India). The drug permeation was assessed as steady-state flux (*fss*) in 24 h from the slope of the linear part of the graph. The processed animal skin was incorporated between donor and receptor chambers at a physiological temperature (37 ± 0.5°C). The maximum is determined at the end of the study on α-MNG-PLGA NPs gel, and the α-MNG gel was 79.32 ± 7.91 and 16.88 ± 7.18 µg/cm^2^/h, respectively, as shown in Figure 7A. The *fss* obtained from α-MNG-PLGA NPs gel was significantly higher than the flux obtained from the α-MNG gel (*p* < 0.05). The drug delivery from NPs resulted in higher drug deposition in the target loci by enhancing stability, thereby enhancing the controlled and sustained release of therapeutic delivery. Furthermore, passively transported NPs after crossing epithelial skin barriers shows an enhanced permeation and retention (EPR) effect nearby the tumor microenvironment due to leaky vasculature, causing more apoptosis [55,56,57]. However, low flux from the α-MNG gel was probably due to less drug solubility, dissolution, and permeation through the cutaneous barrier. The carbopol gel increases in-depth penetration of therapeutic by improving contact time and circumventing loss of aqueous phase from the skin surface [58].

#### Drug Estimation across Skin Layer

The concentrations of drugs from α-MNG-PLGA NPs gel retained in various strata of the skin such as stratum corneum (SC), epidermis layer (EL), and dermal layer (DL) were examined by HPLC and indicated as µg/g of skin tissues. The quantity of α-MNG deposited over SC from the α-MNG-PLGA NPs gel and α-MNG gel was 68.02 µg/g and 29.67 µg/g of skin tissue, respectively. Moreover, in EL, the quantities of drug from α-MNG-PLGA NPs gel and α-MNG gel deposited were 302 µg/g, and 51.21 µg/g of skin. Moreover, the DL had α-MNG-PLGA NPs gel and α-MNG gel concentrations of 267.78 and 21.03 µg/g of skin tissue, as shown in Figure 7B. The drug from α-MNG-PLGA NPs gel was significantly deposited in EL and DL compared to that from α-MNG gel (*p <* 0.05). The higher drug deposition from NPs may be attributed to the nanosize of particles, <200 nm, that circumvent the cutaneous barrier and improve physicochemical stability and compatibility with skin tissue. 

### 2.10. Confocal Laser Microscopy

**Figure 8:**
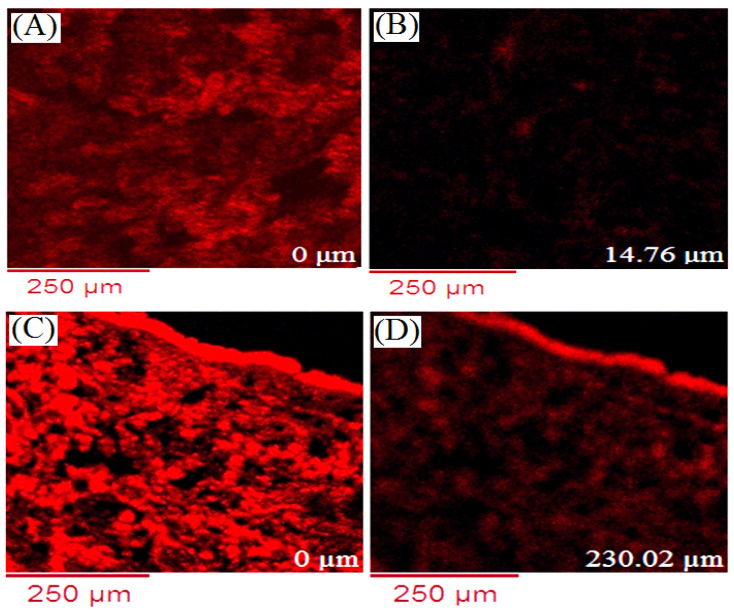
Confocal images of RhB solution (**A**,**B**); and RhB-PLGA NPs (**C**,**D**). Scale bar = 250 µm.

The fluorescent RhB dye incursion transversely to the skin layer was investigated using RhB solution, compared with RhB encapsulated PLGA NPs. The confocal micrograph (Figure 8A,B) revealed that the RhB solution penetrated up to the limited depth inside skin, 15.21 µm, whereas RhB-PLGA NPs (Figure 8C,D) achieved a depth of 230.02 µm inside the layer of skin. The variation in fluorescence intensity in every region of the tissue section indicates a time-dependent process; EL and DL showed more intensity for PLGA NPs than RhB solution. However, the fluorescence intensity of RhB-PLGA NPs and RhB solution at a predetermined time after topical administration showed a time-dependent enrichment of RhB-PLGA NPs in the in different strata of skin and hair follicles. The result indicated higher penetration of RhB from PLGA NPs due to the nanosize of particles (<200 nm) and better skin compatibility of the polymer.

### 2.11. Cell Viability Assay 

**Figure 9:**
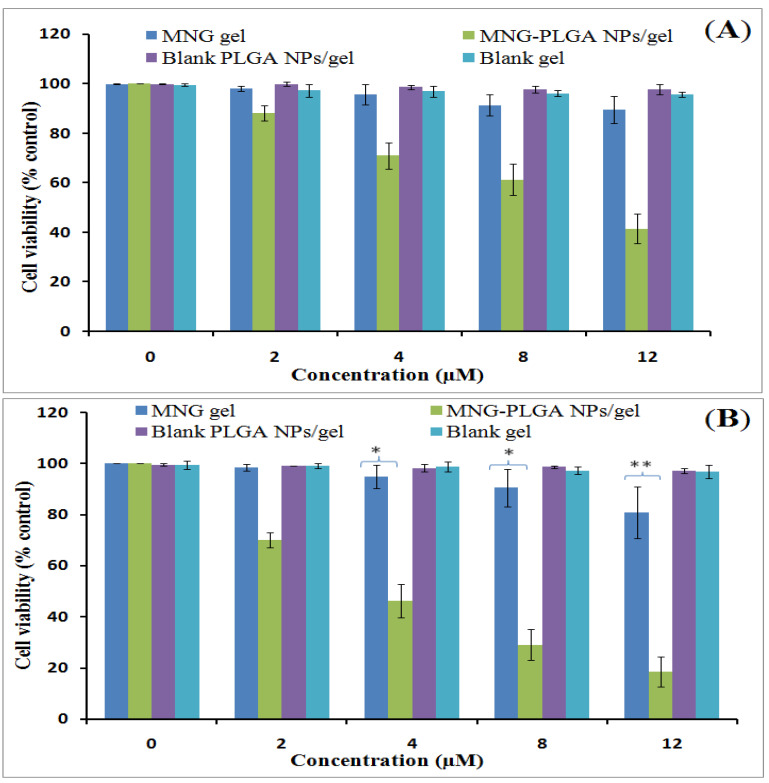
Cell viabilitystudy of α-MNG gel, α-MNG-PLGA NPs gel, blank PLGA NPs gel and blank gel at concentrations, (0, 2, 4, 8, and 12 µM) after incubation times at 24 h (**A**); and after 48 h (**B**) in skin cancer cell line. Data indicated as mean ± SD (*n* = 3); (* *p* < 0.05), (** *p* < 0.01) compared with α-MNG gel.

The MTT assay was performed using α-MNG gel and α-MNG-PLGA NPs gel, both containing an equivalent concentration of the drug, and blank PLGA NPs gel and blank gel to assess the cytotoxic effect of the formulation in comparison to α-MNG gel. The results showed that α-MNG-PLGA NPs gel decreased the viability of the cells in a time- and dose-dependent manner, as shown in Figure 9A,B. At the end of 24 h, the maximum cell viability percentages of treated cells with α-MNG gel and α-MNG-PLGA NPs gel were 89.67% and 41.56%, respectively. After 48 h, the cell viability was 18.50% for α-MNG-PLGA NPs gel and 80.87% for α-MNG gel. The comparative IC_50_ of α-MNG-PLGA NPs gel vs. α-MNG gel was estimated to be 10.00 ± 6.70 and 55.53 ± 6.70 µM after 24 h; 32.65 ± 3.45 and 5.63 ± 0.45 µM after 48 h of incubation in cell line. The observation demonstrated that α-MNG-PLGA NPs gel was more effective in inhibiting carcinoma cells proliferation and thus significantly enhanced apoptosis compared to α-MNG gel (*p* < 0.05). This significant cytotoxicity can be attributed to the high concentration of α-MNG released from PLGA NPs and passively transported inside the cells, thus enhancing the cytotoxic effect. The passive targeting strategy-mediated release of phyto active from the polymeric core could stabilize the therapeutics from protein fraction in blood component and, therefore, enhance the antiproliferation efficacy against melanoma cells [59]. Furthermore, the polymer releases drugs intracellularly through distinguished transporters located in the extracellular matrix of the cell membrane. The cell viability results were consistent with those in previous studies [60,61].

### 2.12. In Vitro Antioxidant Activity

**Figure 10:**
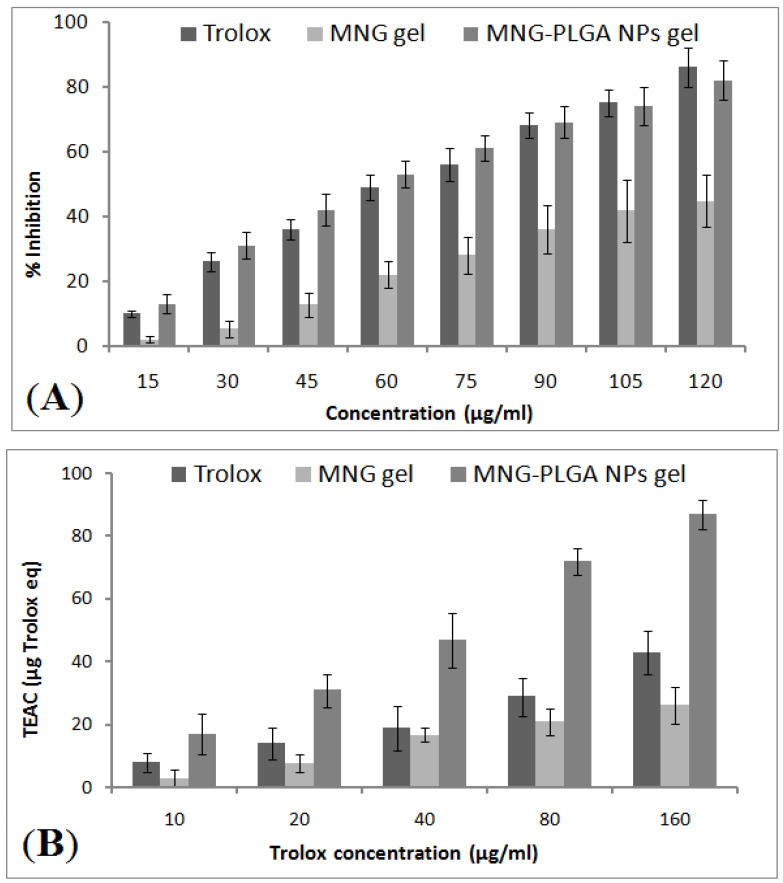
The DPPH antioxidant assay of α-MNG gel and α-MNG-PLGA gel. Antioxidant inhibition effect in percentage (**A**), Trolox equivalent antioxidant activity (TEAC) of α-MNG-PLGA gel compared with trolox and α-MNG gel (**B**). Values indicated as means ± SD (*n = 3*) analyzed by one way ANOVA with Bartlett’s test for statistical significance (*p <* 0.05).

DPPH assay was used to estimate the antiradical scavenging activity of α-MNG gel and α-MNG-PLGA NPs gel. Trolox equivalent antioxidant capacity (TEAC) was calculated from a standard curve of Trolox. The TEAC values of optimized α-MNG-PLGA NPs gel were 17, 31, 47, 72, and 87 µg Trolox equivalent per 10, 20, 40, 80, and 160 µg/mL of Trolox in DPPH assay, respectively (Figure 10A,B). The IC_50_ values reported were 32.44, 48.83, and 100.29 for α-MNG-PLGA NPs gel, standard Trolox, and α-MNG gel, respectively. The antioxidant activity of α-MNG-PLGA NPs gel was significantly higher than that of α-MNG gel (*p* < 0.05).

### 2.13. Stability Studies

**Figure 11:**
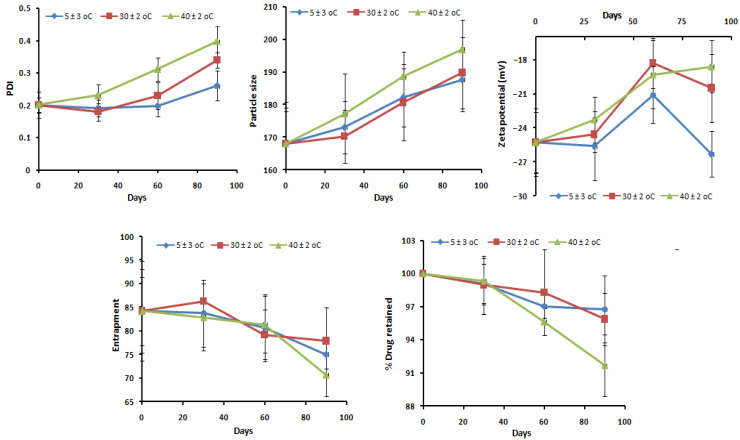
Three-month stability assessment profile of α-MNG-PLGA NPs gel in varying temperature (5 ± 3 °C, 30 ± 2 °C, and 40 ± 2 °C) condition.

A stability experiment studied for three months and a regular assessment at an interval of 30 days concluded that there is an insignificant alteration in the average PS, PDI, zeta potential, % drug entrapment, and % drug retained, which further indicates that the optimized formulation was stable within the specified period. The graphical presentation of the stability assessment profile of α-MNG-PLGA NPs gel under different experimental conditions is expressed in Figure 11.

## 3. Conclusions

The α-MNG-PLGA NPs were formulated using the emulsion–diffusion–evaporation technique and optimized successfully by applying a 3-level 3-factors Box–Behnken design. The α-MNG-PLGA NPs formulations compared well with the α-MNG gel, where the characterization parameters were consistent, noninteracting, and substantially suited to the study. The optimized formulation showed uniform particle distribution, good EE, and stability of the formulation. The selected factors successfully resulted in a positive impact on dependent variables by quadratic equation and well defined the in vitro characterizing fate of α-MNG-PLGA NPs. The analytical studies DSC and FT-IR corroborated that the distinct peak of a drug was also observed in polymeric NPs, which further validated that α-MNG is physicochemically stable and remains encapsulated inwardly to the polymer core of the nanoparticle. The drug release study of the formulation showed initial burst releases abiding by sustained drug release, and the drug release mechanism followed was non-Fickian diffusion of the Higuchi model. The developed formulation showed excellent flux across the skin layer in the skin permeation study, and skin probe fluorescent dye in confocal microscopy revealed significant penetration of NPs into the skin. MTT assay showed significant cytotoxicity of α-MNG-PLGA NPs gel in B16-F10 cell compared to α-MNG gel. Moreover, the radical scavenging activity of α-MNG in α-MNG-PLGA NPs gel showed a marked antioxidant effect compared to that in the α-MNG gel. Thus, α-MNG-PLGA NPs gel is a promising candidate in treating skin cancer and other skin disorders.

## 4. Materials and Methods

### 4.1. Materials

α-MNG (MW 410.46 g/mol; purity = 98%) was obtained from Sigma-Aldrich. PLGA [poly(D, L-lactide-co-glycolide)] lactide: glycolide (50:50) (Mw = 44,628 g/mol; viscosity, 0.71–1.0 dl/g) (Merck, India). Polyvinyl alcohol (PVA) was obtained from Sisco Laboratory (Mumbai, India) and Rhodamine B (RhB) from Sigma-Aldrich (St. Louis, MO, USA). The laboratory solvents, namely acetone, dichloromethane, acetonitrile, and deionized water, were procured from SD Fine Chemicals (Mumbai, India). LC-MS grade reagents, namely acetonitrile, methanol, ammonium acetate, ethyl acetate, glacial acetic acid, and water, were purchased from Sigma-Aldrich (St. Louis, MO, USA). The analytical grades of buffer reagents, i.e., phosphate-buffered saline (PBS), sodium dihydrogen phosphate, sodium hydroxide, disodium hydrogen phosphate, potassium dihydrogen phosphate, and ethanol, were procured from Central Drug House (New Delhi, India). For HPLC analysis, double distilled water was filtered through a micron-sized membrane filter (pore size, 0.45µ) and membrane filter (Durapore pore size, 0.21 µ) using an injectable needle. 

### 4.2. Fabrication of α-Mangostin-Loaded PLGA Nanoparticle

**Figure 12:**
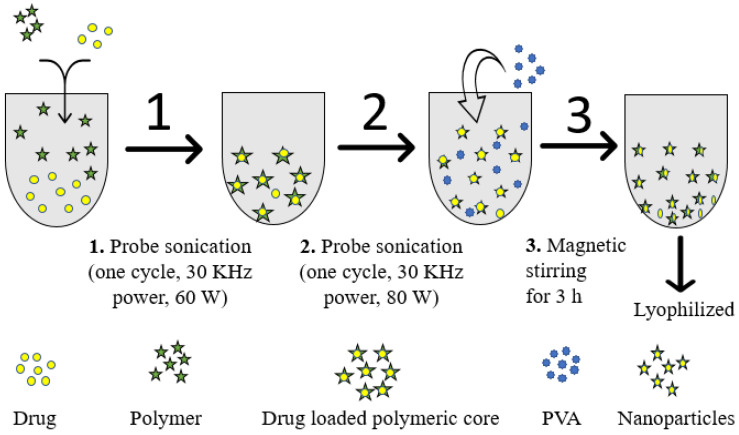
The schematic illustration of preparation of α-MNG-PLGA NPs.

As per our previously reported work, the sonication-tailored α-MNG NPs was constructed using the emulsion–diffusion–evaporation technique [30]. α-MNG (10 mg) and PLGA (2.5% *w/v*) were incorporated in organic solution, dichloromethane (2 mL), with sonication (one cycle, 30 kHz power, 60 W) to form the polymer-encapsulated α-MNG core. Then, the α-MNG polymeric solution was added to the surfactant solution (PVA, 1.5% *w/v*) at a slow flow rate (0.5 mL/min) and emulsified using a sonicator (Probe Sonicator, Hielscher Ultrasonics, Berlin, Germany) underneath an ice bath (one cycle, 30 kHz power, 80 W) to form nanodroplets. These nanodroplets were subjected to continuous magnetic stirring for 3 h, allowing evaporation of the organic phase, and the nanoparticulate suspension was dried overnight to harden the formed NPs. The NPs were separated using a cooling centrifuge (Optima^TM^ LE-80K Ultracentrifuge) at 10,000× *g* for 30 min. Furthermore, the particles were washed thrice and separated from the unentrapped drug and stored in a freezer [39]. The preparation of α-MNG-PLGA NPs is outlined in Figure 12.

### 4.3. Analytical Method

α-MNG content was quantified using the liquid chromatography-mass spectrometry (LC-MS) method. The column configuration was Agilent Zorbax^®^ SB C_18_ (3.5µm; 150 × 3 mm) with guard cartridge C_18_ (4.2 × 2.2 mm). The analyte was separated in isocratic mode using a mobile phase combination of water, ammonium acetate, formic acid (0.1% *v/v*), and methanol at a 300 µL/min solvent flow rate. 

### 4.4. Formulation Optimization

The Box–Behnken design was employed with three factors, three levels, and a total of 17 runs of the experiment using the Design-Expert software (Version 10; Stat-Ease Inc., Minneapolis, Minnesota) [28,29]. The levels of variables employed in the study are indicated in Table 1. The effects of concentrations of independent variables, that is, polymer (X1), surfactant (X2), and ST (X3), were examined on the responses, that is, PS (Y_1_), PDI (Y_2_) and % EE (Y_3_), for the development of NP, as shown in Table 2. The second-order quadratic equations deduced from the design of the experiment for various responses are shown in Table 3. The multivariate linear regression equation generated, which details the relationship among independent and dependent variables, is as follows:

Y = Ao+ A_1_Z_1_ + A_2_Z_2_ + A_3_Z_3_ + A_12_ Z_1_Z_2_ + A_23_Z_2_Z_3_ + A_13_Z_1_Z_3_ + A_11_Z_12_ + A_22_Z_22_ + α_33_Z_32,_ (4)

where Y indicates the measured responses of factors conflated with the level of each factor. Ao indicates the intercept and A_1_ to A_33_ are regression coefficients of the respective variables, while Z_1_, Z_2_, and Z_3_ are the coded levels of independent variables. Z_1_Z_2_, Z_2_X_3_, Z_1_Z_3_, and Z_l2_ (l = 1, 2, 3) show the interaction and quadratic impacts. The levels used in the experimental (base) design are low, medium, high, axis, and central point. After statistical analysis, the best-fitting model within various models (linear, quadratic, cubic, and 2FI) was taken. The F-value showed the best-fitted model; model fitting is shown by PRESS-value, and the lack of fit for the proposed model indicates that it is insignificant. 

### 4.5. Nanoparticle Characterization 

#### 4.5.1. Particle Sizes and Their Distribution

The size of α-MNG-PLGA NPs and their distribution were analyzed using Zetasizer Nano ZSP (Malvern Instruments, Worcestershire, UK) based on a dynamic light scattering procedure. In brief, α-MNG-PLGA NPs (~2 mg) were diluted with deionized water (50 mL); and after sonication, 10 µL of sample volume was applied over the carbon-surfaced copper grid and then was negatively stained with 1% phosphotungstic acid. The studies were performed in triplicate (*n* = 3).

#### 4.5.2. Drug Entrapment and Loading 

The entrapped α-MNG and RbB in the polymeric dispersion were estimated before lyophilization, as previously reported, with minor modification [62]. The NPs were ultracentrifuged at high speed (10,000× *g* at 4 °C for 30 min) (REMI Cooling Centrifuge, India). The aliquots of both α-MNG-PLGA NPs dispersion and RhB-PLGA NPs were separated and subjected to estimation of α-MNG and RbB content using RP-HPLC. The drug entrapment efficiency (% EE) and drug loading were determined as follows:

$$\%\;EE=\frac{\mathrm{cumulative}\;\mathrm{quantity}\;\mathrm{of}\;\alpha -\mathrm{mangostin}\;-\;\mathrm{quantity}\;\mathrm{of}\;\alpha -\mathrm{mangostin}\;\mathrm{in}\;\mathrm{the}\;\mathrm{supernatant}\;}{\mathrm{cumulative}\;\mathrm{quantity}\;\mathrm{of}\;\alpha -\mathrm{mangostin}}\times 100$$

$$\%\;drug\;loading=\frac{\mathrm{cumulative}\;\mathrm{quantity}\;\mathrm{of}\;\alpha -\mathrm{mangostin}\;-\;\mathrm{quantity}\;\mathrm{of}\;\alpha -\mathrm{mangostin}\;\mathrm{on}\;\mathrm{surface}\;\mathrm{layer}\;}{\mathrm{cumulative}\;\mathrm{weight}\;\mathrm{of}\;\alpha -\mathrm{mangostin}\;\mathrm{NPs}\;}\times 100$$

#### 4.5.3. Transmission Electron Microscopy

The PS of the α-MNG-PLGA NPs was studied using transmission electron microscopy (TEM) (Techni TEM 200 Kv, Fei, Electron optics). The α-MNG NPs (1 mg/mL) sample were dispersed in distilled water and bath sonicated. The diluted NPs were disseminated onto the porous film grid and then dried for 10 min, and microscopy was executed at 100 kV, and the image was captured.

#### 4.5.4. Infrared Spectroscopy

Fourier transform-infrared spectroscopy (FT-IR) of plain α-mangostin and α-MNG-PLGA NPs was assessed utilizing an FT-IR spectrometer (BRUKER Corporation, Billerica, MA, USA). The weighed quantity of plain drug and drug-loaded PLGA NPs ~ 5 mg was incorporated in direct contact with a light beam, and the spectrum was recorded in the scanning range of 4000–400 cm^−^^1^.

#### 4.5.5. Differential Scanning Calorimetry

The changes in the enthalpy of plain α-mangostin and α-MNG-PLGA NPs were described by differential scanning calorimetry (Pyris 4 DSC, Perkin Elmer, Waltham, MA, USA). The samples (5 mg) were transferred in aluminum pans, and on the other pan, the reference standard was placed. Both pans were heated simultaneously at a scanning rate of 20 °C/min (20–250 °C) using dry nitrogen gas as effluent gas.

#### 4.5.6. Drug Release and Kinetics Studies

The in vitro drug release was executed for α-MNG-PLGA NPs and compared to α-MNG dispersion. The weighed quantities of NPs, 10 mg of α-MNG, and an equivalent dose α-MNG dispersion were enclosed in a dialysis bag (MW; 8–12 kDa; (Repligen, Waltham, MA, USA) with its ends fixed with a clip. Then, they were transferred to a beaker containing 100 mL of preheated (37 ± 0.5 °C) medium of PBS of pH values of 7.4 and 6.5, which was kept under gentle agitation at 100 rpm [63]. The dialysis bag was activated before the release study under treatment with PBS solution. At predetermined time intervals (0, 4, 8, 12, 16, 20, and 24), 0.5 mL of supernatant was taken from the dissolution medium and filtered using a Durapore^®^ membrane filter of pore size 0.21 µ, using an injectable syringe. The withdrawn medium volume was supplanted by the new medium, so the sink condition is ascertained. The drug concentration was analyzed using RP-HPLC. Moreover, the optimized α-mangostin formulation was fitted to kinetic release models, such as Higuchi, Korsmeyer–Peppas, Hixson–Crowell, first-order, and zero-order model, using the graphical method. The model with good data fit was predicted.

#### 4.5.7. Preparation of Gel

The previously reported technique was employed to prepare the gel base (Kausar et al., 2019). The carbopol 934 (1.0% *w/w*) was magnetically stirred in distilled water (10 mL) for 2 h. In addition, propylene glycol, methyl- and propyl paraben (qs), and triethanolamine (qs) were incorporated with undisturbed stirring until a transparent clear gel was formed. Furthermore, α-MNG-PLGA NPs and α-MNG dispersions were uniformly thrown into the preformed base of gel with uninterrupted stirring. Moreover, the characteristics of the developed gel were analyzed using a gel texture analyzer (TA.XT Plus Texture Analyzer, Stable Micro Systems Ltd., Surrey, UK).

##### Gel Characterization

The pH of the developed gel was measured using a pH meter, and the viscosity was assessed using a Brookfield viscometer. Gel (50 mL) was transferred into a container therein; a spindle 64 was inserted and rotated at a predetermined time; the speed and viscosity of the gel were recorded. The spreadability of the gel was evaluated by placing 500 mg of both α-MNG-PLGA NPs gel and α-MNG gel separately between the glass slides up to a diameter of 2 cm. After that, the gels (500 mg) were placed on the upper glass slide for 5 min and the gel spreading capacities were determined.

#### 4.5.8. Ex Vivo Skin Permeation Studies

The skin penetration capability was investigated using Franz diffusion cell apparatus, having an effective surface area of 0.750 cm^2^ using Logan’s assembly. The rat’s skin was excised, and the fatty layers were surgically removed, rinsed with ethanol, and refrigerated at −80 °C. The skin specimen was mounted safely on the Franz diffusion cells between the donor and receptor compartments with SC cladding towards the donor compartment. Firstly, 7.5 mL of PBS was added to the receptor compartment and agitated at every point using a small magnetic bead at approximately 500 rpm, with the temperature being maintained at 37 ± 0.5 °C. Then, 1 mL of the formulation [α-MNG-PLGA NPs gel or α-MNG gel] was placed on the donor compartment over the skin surface, and samples were taken at fixed intervals for 24 h from the receiver compartment, and the same quantity was replaced with fresh medium. Analysis of the drug content was conducted using RP-HPLC analysis.

#### 4.5.9. Drug Concentration Estimation in Skin Strata

The mounted skin collected from the Franz diffusion cell was washed and cleaned with PBS to remove any adhered particles and was used to estimate the amount of drug present in the various skin strata. The outer SC was removed from the dermis using the tape-stripping method with a scotch crystal tape and sterile surgical scalpel [64,65]. The solution of tissue protein extracting reagent (T-PER) with a tissue: T-PER of 1:10 *w/v* ratio was used to improve the extraction from the skin and probe sonicated for 5 min. Furthermore, the tap strips, epidermis, and dermis were transferred into alcohol, then sonicated to extract the drug, and estimated by UV-spectrophotometry.

#### 4.5.10. Confocal Microscopy

Confocal microscopy was performed to examine the α-MNG distribution and penetration from NPs formulation into various skin layers. In this study, a fluorescent dye, RhB, has to be entrapped into PLGA NPs in place of the drug. During the preparation of NPs using the emulsion–diffusion–evaporation technique, 0.02% *w/v* of fluorescent dye solution was loaded into the formulation, labelled RhB-loaded PLGA NPs. The processed animal skin was mounted onto the Franz diffusion cell on the donor compartment, where the skin SC confronted the donor compartment. A 1 mL of RhB-loaded PLGA NPs was placed on the donor compartment and probe dye release behavior from NPs in the receptor compartment was investigated for 24 h, as shown in the skin permeation study. The temperature of the receptor compartment was maintained at 32 ± 0.5 °C after being filled with 6 mL of PBS, pH 7.4. After the complete study, the skin specimen was removed, gently wiped with deionized water, and prepared on a glass slide. Therefore, it was studied under the confocal microscope set at 540 nm and 630 nm for excitation (λex) and emission wavelength (λem) using an argon laser beam and 65× objective lens (EC-Plan Neofluar 65×/01.40 Oil DICM27). The fluorescent dye, RhB release, and distribution pattern of NPs into distinguished layer of skin was compared with those of plain RhB solution. The confocal microscope optically scanned through z-axis and analyzed the fluorescent distribution and permeation in various layers of skin.

#### 4.5.11. Cell Viability Study

MTT assay was used to study the cytotoxicity of the α-MNG-PLGA NPs gel and α-MNG gel, which was carried out in B16-F10 melanoma cells. The assay shows the transformation of tetrazolium (a yellow color salt) into an insoluble purple color formazan crystal inside the viable cells by a mitochondria enzyme, and the succinate dehydrogenase. About 1×10^6^ cells/well were seeded in each well of the 96-well plate and left overnight. Then, a series α-MNG gel and α-MNG-PLGA NPs gel concentrations (0, 2, 4, 8, and 12 µM) were added to the cells in each well and cultured at 37 °C for 24 and 48 h in a humidified 5% CO_2_ incubator. At the end of each culture period, the culture medium was removed, and the MTT reagent (5 µL) was added to each well for 3 h. After that, the plates were returned to the CO_2_ incubator for 3 h. Then, the insoluble crystals formed were solubilized with dimethyl sulphoxide (DMSO) (130 µL/well), and the optical density (OD) at 570 nm was measured using a microplate reader (Bio-Rad, Des Plaines, IL, USA) [66,67]. Untreated cells or cells exposed to blank PLGA NPs/ gel or blank gel was used as controls (100 % viability). The IC_50_, which is the concentration of the drug that kills 50% cell proliferation compared to control cells, was determined from the cell viability graph using the methods listed in [68]. The cell viability (%) was estimated as mean viability (%) ± standard deviation (SD) (*n* = 3) and calculated by the following:

% Cell viability = Absorbance_treated_/Absorbance_controlled_ × 100. (5)

#### 4.5.12. Free Radical Scavenging Activity

The radical scavenging power of α-MNG in optimized α-MNG-PLGA NPs gel and α-MNG gel was estimated using 2, 2-diphenyl-2-picrylhydrazyl (DPPH) assay as previously reported with minor modification [69]. A 40 µL of α-MNG-PLGA NPs gel and α-MNG gel was mixed with freshly prepared DPPH reagents (220 µL, 0.1 mM), followed by vigorous shaking and incubation for 30 min at 28 °C. OD was then measured using UV-spectrophotometry at λ_max_ of 517 nm. The DPPH antioxidant capacity of α-MNG in the optimized α-MNG-PLGA NPs gel and α-MNG gel was measured as % inhibition in DPPH and estimated as milligrams of Trolox equivalent (TEAC) per gram of sample. The quashed concentration of DPPH was calculated from the standard curve of standard Trolox, and the assay was performed in triplicate (*n =* 3).

The % radical scavenging activity is determined using the following equation:

% Inhibition by DPPH assay = Ao − A1/Ao × 100,(6)

where Ao is the blank; A1 is the sample absorbance calculated using milligram of Trolox equivalent per gram dry extract (mg TE/g extract). IC_50_ (μg/mL) was estimated from the dose–response curve of linear regression analysis, and the concentration calculated enabled achieving 50% of maximum scavenging effect by DPPH radical [70].

#### 4.5.13. Stability Study

The stability study was performed on the optimized α-MNG-PLGA NPs gel as per the International Conference on Harmonization (ICH) guidelines protocol for three months. During the study period, the formulation was monitored for NPs size distribution, PDI, surface charge, drug entrapment, and % drug retained. For stability study, α-MNG-PLGA NPs gel was transferred in a stability chamber at a refrigerated temperature (5 ± 3 °C), at an ambient temperature (30 ± 2 °C/65 ± 5 % RH), and at an increased temperature of 40 ± 2 °C/75 ± 5% RH for three months; the sample was analyzed at intervals of 0, 30, 60 and 90 days [45]. The experiments were performed in triplicate (*n =* 3).

#### 4.5.14. Statistical Analysis

The analysis was performed using ANOVA and Duncan’s multiple range test (DMRT) using the Statistical Package of Social Sciences (SPSS), version 11.0, for Windows. The values were expressed as mean (*n* = 3) ± SD for at least three samples. Level of significance was *p* < 0.05.

## Figures and Tables

**Table 1:** The independent variables and their levels low (0), medium (−1), and high (+1) used in optimization α-MNG-PLGA NPs using experimental design.

Independent Variables	Level Used (Coded)		
	Low (−1)	Medium (0)	High (+1)
X1: Polymer concentration (% *w*/*v*)	2.5	3.00	3.50
X2: Surfactant concentration (% *w*/*v*)	1.00	1.75	2.50
X3: Sonication time (min)	4	8	12
Dependent variables			
Y_1_: Particle size (nm)	Minimize		
Y_2_: PDI	Minimize		
Y_3_: Entrapment efficiency (%)	Maximize		

**Table 2:** The analyzed responses in experimental design for the preparation of α-MNG-PLGA NPs.

Formulation Code	Independent Variables			Responses		
	X1 (% *w*/*v*)	X2 (% *w*/*v*)	X3 (min)	Y_1_ (nm)	Y_2_	Y_3_ (%)
* NP1	3.00	1.75	8.00	176 ± 19.04	0.205 ± 0.02	78 ± 8.67
NP2	2.50	1.00	8.00	187 ± 24.12	0.302 ± 0.03	68 ± 6.12
* NP3	3.00	1.75	8.00	171 ± 15.67	0.210 ± 0.04	79 ± 9.34
NP4	3.50	1.75	12.00	167 ± 13.45	0.233 ± 0.02	83 ± 7.98
* NP5	3.00	1.75	8.00	172 ± 21.81	0.206 ± 0.01	74.9 ± 6.80
NP6	3.00	2.50	12.00	125 ± 10.01	0.276 ± 0.05	75 ± 10.07
NP7	2.50	1.75	4.00	230 ± 33.76	0.281 ± 0.01	51 ± 9.23
NP8	3.50	1.00	8.00	172 ± 11.93	0.215 ± 0.03	83 ± 9.65
NP9	3.00	2.50	4.00	198 ± 19.62	0.306 ±0.04	72 ± 11.92
NP10	2.50	2.50	8.00	167 ± 17.12	0.321 ± 0.02	53 ± 4.47
* NP11	3.00	1.75	8.00	173 ± 16.02	0.199 ± 0.01	76 ± 11.21
NP12	3.50	2.50	8.00	162 ± 14.03	0.268 ± 0.02	90 ± 12.76
NP13	3.00	1.00	4.00	194 ± 24.98	0.203 ± 0.07	74 ± 9.67
NP14	3.50	1.75	4.00	174 ± 17.54	0.222 ± 0.09	81 ± 8.78
NP15	3.00	1.00	12.00	166 ± 19.32	0.312 ± 0.06	84 ± 7.43
* NP16	3.00	1.75	8.00	172 ± 21.78	0.212 ± 0.03	77 ± 9.56
NP17	2.50	1.75	12.00	132 ± 12.67	0.321 ± 0.05	61 ± 7.12

X1: Polymer concentration (% *w*/*v*); X2: Surfactant concentration (% *w*/*v*); X3: Sonication time (min); Y_1_: Particle size (nm); Y_2_: PDI; Y_3_: Entrapment efficiency (%). * Replicas.

**Table 3:** Regression analysis summary for responses particle size (PS; Y_1_)_,_ polydispersity index (PDI; Y_2_), and entrapment efficiency (EE; Y_3_) for fitting into quadratic equation.

Response Surface Quadratic Model	R-Squared	Adj R-Squared	Pred R-Squared	Adeq Precision	PRESS	% CV	Mean	SD
Response 1 (Y_1_)	0.9970	0.9940	0.9853	79.146	128.59	1.04	172.82	1.81
Response 2 (Y_2_)	0.9942	0.9867	0.9476	28.134	1.874 × 10^−3^	2.16	0.25	5.457 × 10^−3^
Response 3 (Y_3_)	0.9935	0.9852	0.9799	39.150	36.26	1.74	74.11	1.29
PS = +173.16 – 5.12 × X1 – 8.37 × X2 – 25.75 × X3 + 2.50 × X1 × X2 + 22.75 × X1 × X3 – 11.25 × X2 × X3 + 2.14 × X1^2^ – 2.86 × X2^2^								
PDI = +0.21 – 0.036 × X1 + 0.017 × X2 + 0.016 × X3 + 8.500E − 0.03 × X1 × X2 – 7.250 – 0.03 × X1 × X3 – 0.035 × X2 × X3 + 0.03 × X1^2^ + 0.040 × X2^2^ + 0.028 × X3^2^								
EE = +76.98 + 13.00 × X1 − 2.37 × X2 + 3.13 × X3 + 5.50 × X1 × X2 − 2.00 × X1 × X3 – 1.75 × X2 × X3 − 5.37 × X1^2^ + 1.88 × X2^2^ − 2.61 × X3^2^								

**Table 4:** Composition, experimental vs. predicted value with percentage error of optimized α-MNG-PLGA NPs.

Variables	Optimum Composition	Response	Observed Value of Response	Predicted Value of Response	Percentage Error
X1	3.39 % *w*/*v*	Y_1_	168.06 ± 17.02	150.87	11.39
X2	1.82 % *w*/*v*	Y_2_	0.201 ± 0.01	0.214	−6.07
X3	8.79 min	Y_3_	84.26 ± 8.23	79.16	6.44

Predicted error (%) = (observed value − predicted value)/predicted value×100%. X1: Polymer concentration (% *w/v*); X2, Surfactant concentration (% *w/v*), X3: Sonication time; Y_1_: Particle size (nm), Y_2_: PDI, Y_3_: Entrapment efficiency (%).

## Data Availability

The data presented in this study are available in article.
